# A shrapnel migration from a peripheral vein to the right ventricle: case report

**DOI:** 10.1093/ehjcr/ytae491

**Published:** 2024-09-20

**Authors:** Ram Sharony, Liran Statlender, Yaron Shapira, Mordehay Vaturi, Shlomit Tamir

**Affiliations:** Department of Cardiothoracic Surgery, Rabin Medical Center, Beilinson Hospital, Tel Aviv University, Petah Tikva 49100, Israel; Department of General Intensive Care and Institute for Nutrition Research, Rabin Medical Center, Beilinson Hospital, Tel Aviv University, Petah Tikva 49100, Israel; Department of Cardiology, Rabin Medical Center, Beilinson Hospital, Tel Aviv University, Petah Tikva 49100, Israel; Department of Cardiology, Rabin Medical Center, Beilinson Hospital, Tel Aviv University, Petah Tikva 49100, Israel; Department of Diagnostic Imaging, Rabin Medical Center, Beilinson Hospital, Tel Aviv University, Petah Tikva 49100, Israel

**Keywords:** Case report, Shrapnel embolism, Bullet embolism, Foreign body in the heart

## Abstract

**Background:**

Foreign bodies that migrate into the heart may include medical devices dislodged from their original location or, rarely, external particles (shrapnel and other foreign bodies) that penetrate the vein, remain intraluminal, and migrate via the venous blood flow to the right heart. Most reported entry sites of these external foreign bodies were in the torso, thigh, or neck; none of them penetrated through a distal extremity of the body. We report a case where shrapnel was found in the right ventricle (RV) following penetrating injury to the hand.

**Case summary:**

An otherwise healthy 24-year-old man presented with an isolated shrapnel injury to his right hand and forearm from an explosion trauma. Computed tomography demonstrated multiple small metal objects in the forearm, hand, and wrist. Additionally, a 3 × 3.5 mm metal object was found in the RV, consistent with a metal shrapnel embolus from the forearm. Echocardiography indicated the fragment to be in a fixed position within the RV, without any additional pathology.

**Discussion:**

Even shrapnel that penetrates through the hand or forearm may migrate to the heart. In this case, following a multidisciplinary discussion, a conservative approach was recommended based on the following condition: lack of symptoms, small size of the foreign body, no obstruction of venous effluent, low risk of significant embolization to the pulmonary vasculature, absence of fever or endocarditis, no current evidence or risk of valve dysfunction, and no myocardial irritation indicated by arrhythmia. The patient was instructed to avoid magnetic resonance imaging scans.

**Learning points**

- External particles such as bullets, shrapnel, and other foreign bodies (FB) may rarely penetrate the vein, remain intraluminal, and migrate to the right heart.
- Most of the reported entry sites of the FB were in the torso, thigh, or neck, but it may also penetrate trough the peripheral part of the body.
- A small FB that migrated to the right ventricle may be left in place when patient is asymptomatic and no evidence of any cardiac injury, endocarditis, or embolization complication risk are noted. Patient should avoid future magnetic resonance imaging examinations.

## Introduction

Migration of foreign bodies (FBs) into the heart has already been described. It may include medical devices such as internal catheters, stents, and needles that reach the right heart chambers by migration from its original location through the venous system.^1,2^ Additionally, external particles such as bullets, shrapnel, and other FBs may penetrate one wall of the vein, remain intraluminal, and migrate by venous blood flow or by gravitation to a remote site, commonly to the right atrium or right ventricle (RV).^3,4^ Most of the reported entry sites of the bullet or shrapnel were in the torso, thigh, or neck, but, to best of our knowledge, none of them penetrated through a distal part of the body like the forearm.^5^

## Summary figure

**Figure:**
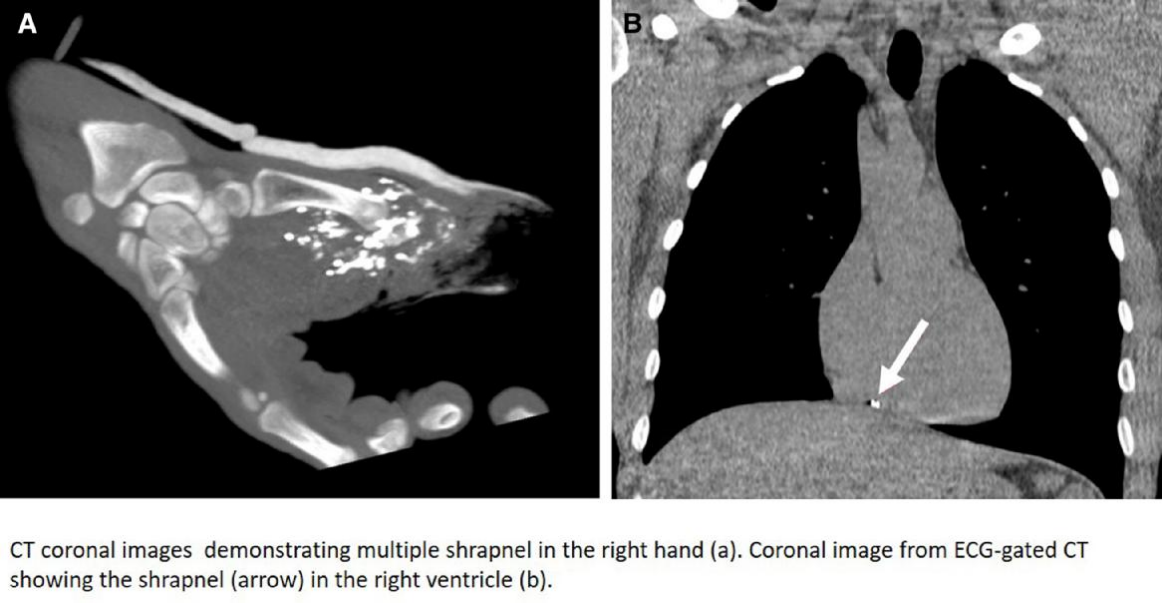


## Case presentation

**Figure 1:**
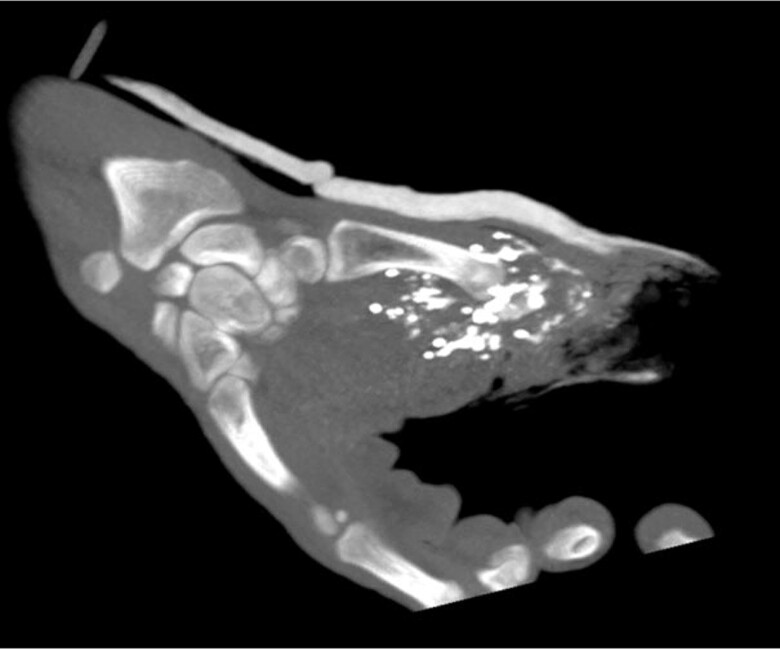
Computed tomography coronal images demonstrating multiple shrapnel in the right hand.

**Figure 2:**
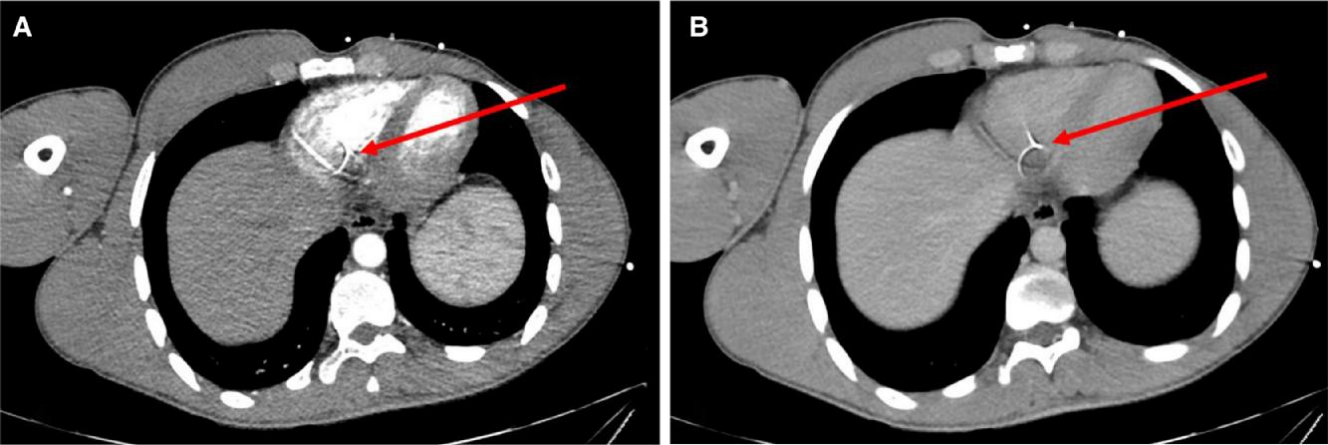
Axial images of arterial (*A*) and venous (*B*) phases of total body computed tomography performed at presentation showing the shrapnel in the right ventricle with adjacent metal artefacts.

**Figure 3:**
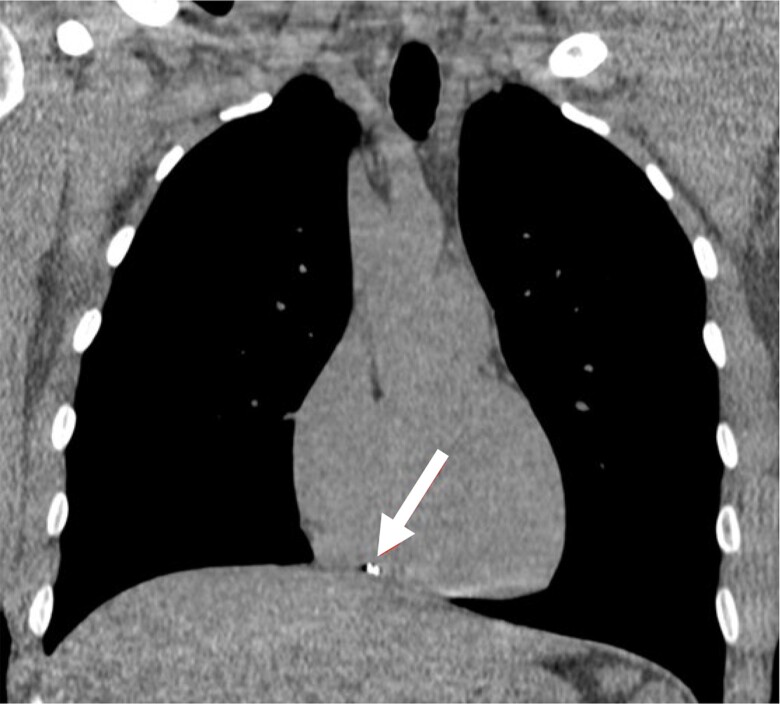
Coronal image from electrocardiogram-gated computed tomography showing the shrapnel (arrow) in the right ventricle.

An otherwise healthy 24-year-old man, without any trauma in his past medical history, presented with shrapnel to his first finger in the right hand from an explosion trauma. Additionally, superficial lacerations were observed in the middle portion of his right forearm and in his face. Physical examination excluded direct injury to his chest or torso. Upon admission to the emergency department, a total body computed tomography (CT) was performed, demonstrating multiple small metal objects in the forearm, most of which were located in the hand and wrist (*Figure 1*) and a few in the forearm. Additionally, a 3 × 3.5 mm metal object was seen in the RV (*Figures 2A* and *B* and *3*); however, no pericardial effusion, pleural effusions, pneumothorax, lung parenchymal lacerations, and subcutaneous haematoma or laceration were demonstrated. Therefore, the FB in his heart was consistent with a metal shrapnel embolus from the forearm to the RV. Echocardiography examination indicated the fragment to be in a fixed intracavitary position within the RV. Neither pericardial effusion nor any valvular pathology, ventricular dysfunction, or evidence of pulmonary hypertension was noted. Echocardiography ‘bubble test’ with Valsalva manoeuvre excluded shunts between the right and left chambers. Doppler examination of his right arm demonstrated normal venous flow pattern. The patient underwent surgical debridement and open reduction of fractured phalanges with internal fixation of his first finger. He was hospitalized for monitoring and observation in the general intensive care unit and later in the department of cardiothoracic surgery. Follow-up non-contrast CT with electrocardiogram gating was performed 5 days after the injury, showing stable location of the FB in the RV, adjacent to the inferior basal wall. A multidisciplinary discussion regarding the treatment options for the FB in the heart was conducted. Based on the following condition,^6,7^ lack of any symptoms, no obstruction of venous effluent, low risk of significant embolization to the pulmonary vasculature, absence of fever or bacterial endocarditis, no risk of valve dysfunction, and no myocardial irritation indicated by arrhythmia, conservative treatment was recommended. The patient was treated with standard prophylactic anti-coagulation (subcutaneous enoxaparin) according to the institutional protocol for trauma patients. The patient was discharged in a good clinical status with a warning against undergoing any magnetic resonance imaging (MRI) examinations. The patient remains asymptomatic after a 4-month follow-up.

## Discussion

This case report reviews a patient who suffered multiple shrapnel injuries to the forearm, one of which, measuring 3 mm, penetrated into the venous system and migrated to the heart, finally lodging in the RV. As compared with the previous reports where the entry site was in a large vessels,^5^ the unique aspect of this case is the fact that the FB (shrapnel) entry site was a minor vein in the hand or mid forearm.

In a large series analysed by Leitman and Vered,^8^ most of the FBs were internal medical devices [inferior vena cava (IVC) filter, ventriculo-peritoneal shunts, different catheters, stents, and wires], while only 11% were non-medical devices, i.e. external FB like needles, staple, pieces of metal, and bullets. Another report from the Vietnam War reported 22 cases of intravascular emboli out of 7500 cases of vascular system penetrating trauma (0.3%), most involving explosive device fragments.^9^ A more recent series from the Global War on Terror (in Iraq and Afghanistan) found four cases of intravascular fragment emboli among 346 patients with penetrating vascular system injuries,^10^ and none of them entered through the distal limbs. Although Liu *et al*.^11^ reported a case where a shrapnel penetrated the proximal third of the upper arm from a piece of metal and migrated after 2 days to the RV, the current report focuses on penetrating site in the forearm and hand, i.e. a peripheral portion of the body. Yoon *et al*.^5^ specified that entry sites of the external FB in all cases were major vessels in the torso, neck, or thigh. None of the 62 reported cases entered through a peripheral limb distal to arm or thigh. The FBs were located in the right atrium (9.7%), RV (54.8%), and pulmonary arterial tree (32.3%).

Two factors play a role in FB migration: blood flow and gravity. The venous blood flow may drift the FB to the right heart and potentially to the pulmonary vasculature. However, gravity may cause a retrograde venous FB embolism, i.e. the migration of a FB against the blood flow. Migration of FB from the right heart into the IVC and subsequently to the renal vein and the hepatic vein has been reported^6^ as well as a bullet injury to the thigh, migrated across the right atrium and eventually lodged in the superior vena cava-azygos vein junction.^12^

The time frame between the FB injury and its migration to the destination site may vary between the acute event diagnosis date to several months after the primary injury. It has been suggested that only 20% of cases have been diagnosed within 24 h since the initial event.^8^

Guidelines do not exist regarding the ideal management of bullet and shrapnel emboli. It may be immediately extracted by trans-catheter approach or even by open heart surgery, but the risk–benefit balance of each approach should be analysed by a multidisciplinary team and finally discussed with the patient. A management algorithm for venous bullet embolism has been suggested^5^ including patient-related factors (clinical condition and operative risk), FB-related factors [size (±5 mm) and shape], and potential damage (location, stability, danger of cardiac perforation, arrhythmia, and valve dysfunction).

Isolated FB in the RV like Micra pacemaker or pacemaker lead does not require anticoagulation. Therefore, we did not recommend full dose anti-coagulation therapy (either warfarin or new oral anti-coagulants) beyond the routine prophylaxis protocol.

According to the European Society of Cardiology guidelines for the management of endocarditis, antibiotic prophylaxis (during dental and other procedures) is not recommended for patient with FB in the RV.

## Conclusion

It should be emphasized that even a small shrapnel penetrating the peripheral part of the body may migrate to the RV through the venous flow. In this case, based on the small size of the FB, lack of evidence of cardiac structure injury, the stability of the object, and low risk of complications, it was recommended to avoid any intervention. Patient should avoid MRI studies.

## Lead author biography


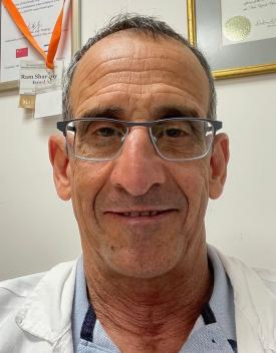


Dr Sharony serves as the “Director of Minimally Invasive Unit Cardiothoracic Surgery” at the Rabin Medical Center, Israel. Dr Sharony specializes in complex and high-risk cardiac surgery as well as complex valve surgery. His clinical and research interests include coronary artery bypass grafting, valvular heart disease, and circulatory support. Dr Sharony has accomplished his fellowship at NYU Langone Medical Center. He is active in scientific, research, and professional communications. He has published over 80 articles and book chapters in the peer-reviewed medical journals and textbooks.

**Consent:** The patient consents publication of this manuscript, complying with the COPE guidelines.

**Funding:** None declared.

## Data Availability

The data underlying this article will be shared on reasonable request to the corresponding author.
